# Altered *O*-glycomes of Renal Brush-Border Membrane in Model Rats with Chronic Kidney Diseases

**DOI:** 10.3390/biom11111560

**Published:** 2021-10-21

**Authors:** Aiying Yu, Jingfu Zhao, Jieqiang Zhong, Junyao Wang, Shiv Pratap S. Yadav, Bruce A. Molitoris, Mark C. Wagner, Yehia Mechref

**Affiliations:** 1 Department of Chemistry and Biochemistry, Texas Tech University, Lubbock, TX 79409, USA; Aiying.Yu@ttu.edu (A.Y.); jingfu.zhao1992@gmail.com (J.Z.); Jieqiang.Zhong@ttu.edu (J.Z.); Junyao.Wang@ttu.edu (J.W.); 2 Department of Medicine, Nephrology Division, Indiana University, Indianapolis, IN 46202, USA; ssyadav@iu.edu (S.P.S.Y.); bmolitor@iu.edu (B.A.M.); wagnerm@iu.edu (M.C.W.)

**Keywords:** *O*-glycan, brush-border membrane, proteinuria and hypertension, obese and diabetic, chronic kidney disease, differential expression analysis, LC–MS/MS

## Abstract

Chronic kidney disease (CKD) is defined as a decrease in renal function or glomerular filtration rate (GFR), and proteinuria is often present. Proteinuria increases with age and can be caused by glomerular and/or proximal tubule (PT) alterations. PT cells have an apical brush border membrane (BBM), which is a highly dynamic, organized, and specialized membrane region containing multiple glycoproteins required for its functions including regulating uptake, secretion, and signaling dependent upon the physiologic state. PT disorders contribute to the dysfunction observed in CKD. Many glycoprotein functions have been attributed to their *N*- and *O*-glycans, which are highly regulated and complex. In this study, the *O*-glycans present in rat BBMs from animals with different levels of kidney disease and proteinuria were characterized and analyzed using liquid chromatography tandem mass spectrometry (LC–MS/MS). A principal component analysis (PCA) documented that each group has distinct *O*-glycan distributions. Higher fucosylation levels were observed in the CKD and diabetic groups, which may contribute to PT dysfunction by altering physiologic glycoprotein interactions. Fucosylated *O*-glycans such as 1-1-1-0 exhibited higher abundance in the severe proteinuric groups. These glycomic results revealed that differential *O*-glycan expressions in CKD progressions has the potential to define the mechanism of proteinuria in kidney disease and to identify potential therapeutic interventions.

## 1. Introduction

Glycosylation is one of the most prevalent post-translational modifications in eukaryotes that impacts protein, lipid, and possibly RNA structure and function [1]. Numerous biological functions such as cell–cell signaling, cell–cell communication, protein targeting, inflammatory response, and immune response are associated with protein glycosylation [2,3,4,5,6]. The importance of protein glycosylation has been demonstrated in the progression of many human diseases, including liver diseases [7,8], neurological diseases, [9] Alzheimer’s disease [10,11], inflammatory diseases [12,13], and cancers [14,15,16,17,18,19]. The pathological conditions caused by these diseases are related to the existence of uncommon glycans or quantitative alterations of *N*-linked or *O*-linked glycans [20]. Glycoproteins with altered *O*-glycan expression have been used as circulating tumor biomarkers and have been shown to trigger reduced PT albumin endocytosis [21,22,23]. It has been reported that *O*-glycan alterations were observed in the diabetic ovarian tissues of mouse and gastric cancer patients compared with healthy controls [24,25].

The renal proximal tubule (PT) is the main site of water, electrolyte, and nutrient reabsorption. The PT has a specialized apical membrane, brush-border membranes (BBMs); this dramatically increases its surface area and contains multiple glycoproteins that contribute to its multiple functions and cell responses, dependent upon the physiologic state [26]. These dynamic functions contribute to maintaining homeostasis and are often disrupted in kidney disease [26,27]. The global prevalence of chronic kidney disease (CKD) is 11.7% to 15.1%, and the number of patients who suffer end-stage kidney disease is between four and seven million [28]. Evidence has shown that the global increase in CKD is mainly associated with an increase in proteinuria, hypertension, obesity, diabetes mellitus, and aging [29,30,31,32]. Many studies have applied genomic, epigenetic, proteomic, and transcriptomic approaches to better define, diagnose, and identify biomarkers for CKD [33,34,35,36]. However, these approaches have focused on protein- and gene-level characterization while the glycome has not been well-documented, and few studies have explored the glycan changes in the PT BBM. The BBM glycoproteins’ conformation, interactions, clustering, and lateral mobility can all be impacted by their glycosylation state. Glycosylation in BBMs of cultured kidney cells has been demonstrated to influence organic cation transport and albumin endocytosis [23,37]. Therefore, a comprehensive investigation of renal BBM glycosylation is necessary to characterize the glycan changes that occur with disease in this dynamic and important membrane. This is an important first step in understanding the role of these changes, identifying which ones are most important for preventing dysfunction and developing potential new therapeutics that could prevent harmful glycan alterations.

*O*-glycans are essential for their protective functions, such as blocking bacterial adhesion, as well as for maintaining the function of the epithelial barrier [38,39]. Their quantitative analysis relies on analytical techniques with high sensitivity and resolution such as mass spectrometry (MS). MS combined with liquid chromatography (LC) has been widely used as a powerful analytical method for comprehensive quantitative glycan analysis capable of complex sample analysis [40]. In this work, we combined several techniques that included an enzymatic/chemical *β*-elimination approach [41], permethylation [42,43], and NanoLC–MS/MS analysis. The *O*-glycomes of BBMs from rats with normal physiology were compared with those with abnormal physiology including proteinuria, hypertension, and hyperglycemia. Since the PT BBM is the principal site for protein uptake and glycans are known to regulate multiple membrane protein functions, it is important to fully understand the dynamic glycan changes that occur as proteinuria increases. Our results show distinct *O*-glycan changes, which will provide a foundation from which more specific questions of glycan regulation and specific modifications to important BBM glycoproteins, i.e., megalin and cubilin, can be explored.

## 2. Materials and Methods

### 2.1. Materials and Reagents

Pronase from Streptomyces griseus, sodium deoxycholate (SDC), iodomethane, dimethyl sulfoxide (DMSO), ammonium bicarbonate (ABC), and sodium hydroxide (NaOH) beads were purchased from Sigma-Aldrich (St. Louis, MO, USA). High-performance liquid chromatography (HPLC)-grade methanol, HPLC-grade water, acetonitrile (ACN), and formic acid (FA) were obtained from Fisher Scientific (Fair Lawn, NJ, USA). Anhydrous ethanol was purchased from Pharmco-Aaper.

### 2.2. Animals

Munich Wistar Frömter (MWF) rats were derived from a colony generously provided by Dr. Roland Blantz (UCSF, San Diego, CA, USA) and maintained in the Indiana University LARC facility. Zucker ZSF1 obese male rats were obtained from Charles River. All rats received water and food ad libitum throughout the study (MWF received standard rat chow, and ZSF1 received LabDiet 5008). All experiments were conducted according to the guidelines of National Institutes of Health for the Care and Use of Laboratory Animals guidelines and were approved by the Animal Care and Use Committee at the Indiana University School of Medicine.

BBM pellets from the kidney cortices of five different groups of rats were used to reveal variations in the *O*-glycan expressions. Groups one to four were Munich Wistar Frömter rats. The reason for choosing Munich Wistar Frömter rats as CKD disease models is that they offer a unique opportunity to follow physiological changes as they age while also enabling direct visualization of glomerular filtration and Proximal tubule cell uptake at the BBM since they contain surface glomeruli. This species has many surface glomeruli and has been studied extensively in micropuncture. It has been reported that the development of hypertension and proteinuria was found in males by week 8, which escalates gradually to ≥400 mg/24 h urinary albumin excretion by week 32, with around 50% sclerotic glomeruli by week 40 [44,45]. Severe proteinuria was observed in the old male group (G3) while only mild proteinuria was observed in the old female group (G1). Groups two (G2) and four (G4) were young female and young male rats, used as healthy controls. For the diabetic model in this study, group five (G5) was Zucker rats from Charles Rivers, which have been widely used as a diabetic model with metabolic syndromes including type 2 diabetes and renal function impairment [46]. Table 1 shows detailed information for the five sample groups. In this study, G1 to G4 contain four biological replicates, while G5 contains six biological replicates. Each was from an individual rat kidney cortex sample and pooling of the rats was not performed.

### 2.3. Isolation of Renal Proximal Tubular Brush-Border Membranes

Rat BBMs were purified using a modification of the procedure by Biber et al. [47], as described previously. In brief, the rats were perfused with saline and the kidneys were removed. The kidneys were kept in cold phosphate-buffered saline during dissection of the cortex. The outer cortex was rapidly frozen in liquid nitrogen, and 1 g of renal cortex tissue was placed in a sample tube and homogenized with 7.5 mL of cold buffer A (300 mM mannitol, 5 mM egtazic acid (EGTA), 12 mM Tris-base, pH 7.1, 1 mM phenylmethylsulfonyl fluoride, and protease cocktail). All cortexes were homogenized using the Polytron tip PTA10S (2–45 s bursts). Next, 10 mL of water and 210 µL of 1 M magnesium chloride (MgCl_2_) were added to the tube, mixed, and placed on ice for 15 min. The tube was then centrifuged at 3000× *g* for 15 min at 4 °C to remove aggregated membranes and incompletely homogenized tissue. The supernatant was decanted into a new centrifugation tube and then centrifuged at 38,000× *g* at 4 °C for 30 min. The pellet was resuspended with 1 mL buffer B (150 mM mannitol, 2.5 mM EGTA, and 6 mM Tris-base, pH 7.1) and centrifuged at 38,000× *g* for 30 min. Two cycles of Mg precipitation were conducted, and the final BBM pellet was resuspended in 1–2 mL of buffer B, aliquoted into microfuge tubes, pelleted, frozen in liquid nitrogen, and stored at −80 °C until analysis.

### 2.4. Protein Extraction

The frozen BBM pellets were thawed at room temperature, 100 µL of 5% SDC buffer was added to the pellets, and the final concentration of the SDC was adjusted to 2.5% by adding 100 µL of the 50 mM ABC buffer. Next, the membrane was broken up by a bead beater (BeadBug microtube homogenizer, Benchmark Scientific, Edison, NJ, USA) at 4 °C. The cycle of the bead beating procedure was 30 s of shaking followed by 30 s intervals, and this cycle was repeated 5 times. After that, the samples were sonicated in an ice water bath for further breaking for 1 h. Next, the samples were taken out of the ice bath for centrifugation at a speed of 1000× *g* for 1 min and then the speed was increased to 14,800 g for 10 min. Finally, the supernatants that contained isolated proteins were collected and stored in new Eppendorf tubes.

### 2.5. BCA Protein Assay and Pronase Digestion

After protein extraction, the proteins were denatured at 90 °C for 15 min, and 2 µL of proteins were used to perform a protein assay with a BCA protein assay kit (Thermo Scientific/Pierce, Rockford, IL, USA). After the protein concentration measurement, samples with a range of 200 to 300 µg of proteins were combined with 50 mM ABC buffer to dilute the SDC to 0.5%. After adjusting the volume, the samples were treated with pronase at 55 °C for 48 h. The ratio of pronase to protein was 1:10 (µg/µg). After incubation, 1% FA was added to the solution to precipitate SDC. The samples were centrifuged at 1000× *g* for 1 min followed by 14,800 g for 10 min. The supernatant was collected and dried using a SpeedVac benchtop vacuum concentrator (Labconco, Kansas City, MO, USA). The dried samples were resuspended with 50 µL of water and subjected to dialysis overnight to remove salts and the remaining SDC using a 100–500 Da molecular weight cutoff dialysis membrane (Spectrum Laboratories, Rancho Dominguez, CA, USA). After dialysis, the samples were dried for the permethylation procedure.

### 2.6. Solid-Phase Permethylation of O-glycans

The dried samples were permethylated using the previously reported solid-phase permethylation method [43] and were dissolved in a buffer that contained 30 μL DMSO and 1.2 μL HPLC water. The reaction columns were loaded with NaOH beads that were suspended in DMSO to half of the total volume, followed by centrifugation at 1800× *g* for 2 min. Next, 20 μL of iodomethane was added to the samples and then vortexed. The mixtures were loaded into the reaction column and incubated for 25 min at room temperature. Then, 20 μL of iodomethane was added to the column and continued to incubate for another 15 min. After incubation, the columns were centrifuged at 1800× *g* for 2 min. A volume of 30 μL of ACN was added to the column to elute the permethylated *O*-glycans. All columns were centrifugated at 1800× *g* for 1 min. The eluents were collected and dried overnight under speed vacuum. Dried permethylated *O*-glycans were resuspended with 20% ACN and 0.1% formic acid for LC–MS/MS analysis.

### 2.7. Instrument Method

The permethylated *O*-glycans were analyzed with an Ultimate 3000 NanoLC system (Dionex, Sunnyvale, CA, USA) coupled with an LTQ Orbitrap Velos mass spectrometer (Thermo Scientific, San Jose, CA, USA). Each injection contained *O*-glycans released from 300 µg of proteins. An Acclaim PepMap 100 C18 trap column (75 µm × 2 mm, 3 µm, 100 Å, Thermo Scientific, Pittsburgh, PA, USA) was used for sample loading and purification. All separations of *O*-glycans were conducted on an Acclaim PepMap 100 C18 column (75 μm × 150 mm, 2 μm, 100 Å, Thermo Scientific, Pittsburgh, PA, USA). Mobile phase A consisted of 98% water, 0.1% FA, and 2% ACN, and mobile phase B consisted of 100% ACN with 0.1% FA. The flow rate was 0.35 μL/min. In the first 10 min of the gradient, mobile phase B remained at 20% and increased to 42% in the next one min. From 11 to 48 min, the percentage of mobile phase B was increased to 55% linearly and elevated to 90% at 49 min. In the next 5 min, the percentage was kept at 90% and then returned to 20% from 55 to 60 min. The LTQ Orbitrap Velos mass spectrometer was set to the positive ion mode. The full MS scan was set to a 100,000 resolution with a 200 to 2000 *m*/*z* mass range. MS^2^ was operated in Data-dependent acquisition (DDA) mode. DDA was used to select the top four most intense precursor ions from the full MS scan for collision-induced dissociation (CID) and higher collisional energy dissociation (HCD) MS^2^ fragmentation. The normalized collision energy for CID was 30% with 10 ms activation time. In HCD, the normalized collision energy was 45% with 0.1 ms activation time. The isolation width was set to 3.0 *m*/*z* in both CID and HCD. Multiple-reaction monitoring (MRM) was conducted on a TS Vantage triple quadrupole mass spectrometer (Thermo Scientific). The MRM LC–MS/MS method has been shown to be a viable strategy for identification and quantification of *N*-glycans [48]. This method depends on the transition ions of analytes that are observed in the tandem mass spectrum. With increased signal-to-noise ratios, the method shows more sensitive quantitation of low abundance structures [49]. In this study, the MRM method was used as a second quantification method to further confirm and quantify *O*-glycan compositions. The parameters for the MRM method were set as follows: the peak width was set at 0.7 FWHM, and the scan time was 0.7 s at a mass range of 200–1500 *m*/*z*. The normalized collision energy was set at 30% to 45%.

### 2.8. Data Analysis

Raw files were processed by MultiGlycan software to detect possible *O*-glycan compositions [50]. This software can provide a more confident glycan annotation by linking together envelopes using multiple adducts or a specified adduct simultaneously. The mass tolerance of the monoisotopic peak of the MultiGlycan set-up was 5 ppm, and the isotope envelope tolerance was 6 ppm. The possible glycan compositions were further identified by manually matching the observed *m*/*z* of the monoisotopic peaks from the extracted ion chromatograms (EIC) from Xcalibur (Thermo Scientific) with the theoretical values. The EIC peaks corresponding to each glycan in different charge states and multiple adduct forms were added to calculate the glycan intensity. The relative quantification of the *O*-glycans from the BBM samples were used to investigate the *O*-glycan expressions [51]. The relative quantification of each identified *O*-glycan was calculated by dividing the individual *O*-glycan peak area by the sum of all detected *O*-glycan peak areas. The relative quantitation values of each *O*-glycan from five sample groups were subjected to MarkerView Software (Sciex) for unsupervised principal component analysis (PCA) to visualize differences among groups. A two-tailed student *t*-test was applied to identify statistically significant *O*-glycan compositions between the disease-free control group and the disease group with additional Bonferroni Correction to reduce type I error.

## 3. Results

### 3.1. O-glycome Profiles of BBMs

Through the efficient enzymatic/chemical approach, followed by permethylation coupled with effective separation and the high sensitivity of the LC–MS method, a total of 59 *O*-glycans were identified and quantified among five groups of BBM samples. All identified *O*-glycans from the five sample groups are listed in the Supplementary Materials Table S1 with their theoretical *m*/*z*, observed *m*/*z*, average relative abundance, standard deviation, and *p*-value. The mass accuracy of all these *O*-glycans was less than 10 ppm. The distribution of the different types of *O*-glycans among the five groups are shown in the Supplementary Materials Table S2. The identifications of all *O*-glycans were confirmed by full MS and MS^2^. One example of *O*-glycan composition identification is shown in the Supplementary Materials Figure S1. *O*-glycan HexNAc_1_Hex_1_NeuAc_2_ (1-1-0-2) was confirmed by the full MS, as shown in the Figure S1a inset. Then, the composition was further validated by the diagnostic fragment ions of the specific positions from the monosaccharides in the MS^2^ spectrum (Figure S1b).

### 3.2. Unsupervised PCA

**Figure 1:**
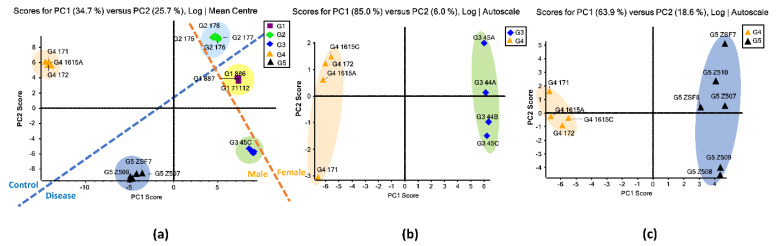
(**a**) Unsupervised PCA of the BBM *O*-glycans from the five sample groups. Symbols with the same color and shape were replicates from same sample group. (**b**) Unsupervised PCA of the BBM *O*-glycans from older males with proteinuria and hypertension (G3) and from the young male control (G4). (**c**) Unsupervised PCA of the BBM *O*-glycans from obese and diabetic male (G5) and from the young male control (G4). G3 were MWF male rats (32-42 weeks), G4 were MWF male rats (7 weeks), and G5 were ZSF1 male rats (16 weeks).

PCA was used for the initial statistical evaluation of the five sample groups. PCA is a multivariate statistical technique that can reduce the dimensionality of large datasets by keeping only the important information that reveals the structure of the observations and the variables. The pattern of similarity of the observations and the variables can be displayed as points on the PCA maps [52]. Figure 1a displays the unsupervised PCA plot for the *O*-glycomic data of five groups of BBM samples. The plot indicates good reproducibility of the replicates, as the data points of replicates were tightly clustered. Moreover, each of the sample groups were separated from one another by the primary component (PC1) and secondary component (PC2). Among these five groups, the group 1 (G1) cluster showed the closest similarity to the group 2 (G2) cluster, which was expected because these two groups were from female rats. In Figure 1a, gender differences are roughly split on either side of the orange line, and the disease-related groups and healthy controls are separated by the blue line. This indicates that distinct differences in *O*-glycan expressions exist between the two genders as well as the disease progression. The female controls (G2) and male controls (G4) showed their clusters separated in the PCA plot, suggesting that there was a variation in *O*-glycan expression between the two controls (Supplementary Materials Figure S3a). When comparing the *O*-glycan expressions between the proteinuria and hypertension male group (G3) and the male control group (G4), PCA clusters of the two groups were separated without any overlap, implying a distinct difference in *O*-glycan expressions between these two groups (Figure 1b). According to the separated clusters in the PCA plots, different expressions were also observed between the older females with proteinuria group (G1) and the female control group (G2) (Supplementary Materials Figure S3b) as well as the obese and diabetic male group (G5) and the male control group (G4) (Figure 1c).

### 3.3. Comparison between the Proteinuria and Hypertension Groups and the Control Group

When comparing the differences in *O*-glycan expressions in the sample groups with proteinuria and hypertension to the control groups, a statistical test was performed using a student *t*-test. Considering that the increase in multiple testing could increase the likelihood of a type I error, the Bonferroni correction was applied to adjust the *p*-values to maintain the statistical power [53]. After a MultiGlycan analysis with an additional manual check, a total of 59 *O*-glycans were identified and quantified across the five sample groups with varying relative abundances. After the *t*-test with Bonferroni correction, *O*-glycans identified with *p*-values less than 0.001 were considered statistically significant.

**Figure 2:**
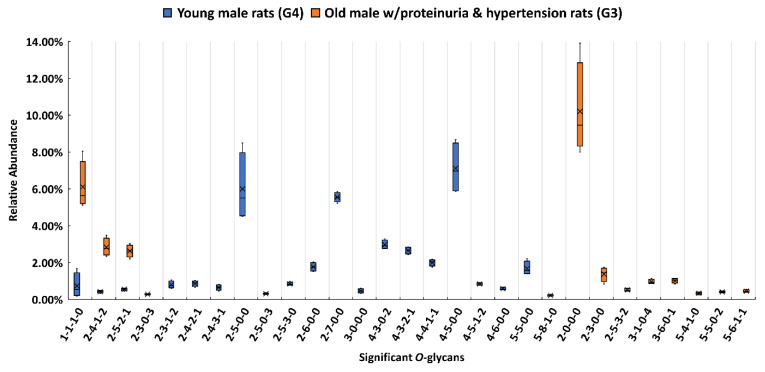
Box plot for the relative abundance of 29 significant *O*-glycans (*p* < 0.001) between young male controls (G4), and older males with proteinuria and hypertension (G3). The putative composition was assigned to each glycan composition. The X axis denotes the four-digit codes for *O*-glycan compositions. The Y axis is the relative abundance. The error bars are the 95% confidence interval. The four-digit codes represent *O*-glycan compositions. X-X-X-X stands for HexNAc-Hexose-DeoxyHex-NeuAc. HexNAc includes *N*-acetylglucosamine and *N*-acetylgalactosamine. Hexose includes galactose, glucose, and mannose. Deoxyhexose is fucose, and NeuAc is *N*-acetylneuraminic acid.

When the relative abundance of each identified *O*-glycan was compared between the proteinuria and hypertension male group (G3) and the male control group (G4), 29 O-glycans showed a statistically significant difference (*p* < 0.001). The relative abundance of the 29 O-glycans between the two groups were submitted to IBM SPSS Statistics to generate a box plot (Figure 2). All *O*-glycans were classified into four major types, which included sialylated, fucosylated, sialylated, and fucosylated, and other types (compositions without sialic acid and fucose). The distributions of the different types of O-glycans among the five groups are displayed in the five pie charts shown in the Supplementary Materials Figure S2. To validate the statistically significant *O*-glycans, their abundances were also compared using the targeted MRM method. The transitions and optimized collision energy of *O*-glycans to conduct an MRM experiment are listed in the Supplementary Materials Table S3. For the sialylated compositions, 3-1-0-4, 3-6-0-1, and 5-5-0-2 were only found in the G3, while 2-5-0-3 and 4-3-0-2 only existed in G4, as shown in Figure 2. For the fucosylated compositions, 1-1–1-0 was upregulated in G3 compared with G4, 5-4-1-0 was only detected in G3, while 2-5-3-0 and 5-8-1-0 were only observed in G4. In the sialylated and fucosylated types, compositions 2-4-1-2 and 2-5-2-1 were observed to be upregulated in G3; 5-6-1-1 was only expressed in G3; and 2-3-1-2, 2-4-2-1, 2-4-3-1, 4-3-2-1, 4-4-1-1, and 4-5-1-2 were only detected in G4. For compositions without sialic acid and fucose, 2-0-0-0 showed the highest relative abundance and 2-3-0-0 was only found in G3. However, 2-5-0-0, 2-6-0-0, 2-7-0-0, 4-5-0-0, and 4-6-0-0 were only found in G4. Different expressions of all of these significant compositions were consistent with the MRM results shown in the Supplementary Materials Table S3. Since the comparison was made within the same gender (male), the bias from gender was minimized and the significant expression of the glycan was related more to proteinuria and hypertension.

**Figure 3:**
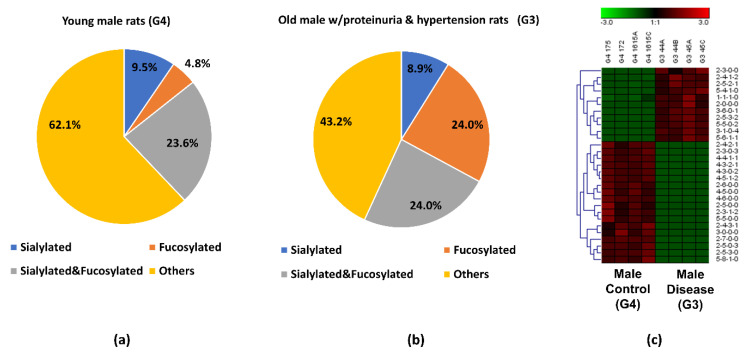
The distribution changes of different *O*-glycan types from (**a**) young male control (G4), and (**b**) older male with proteinuria and hypertension (G3); (**c**) heatmap of 29 *O*-glycans that exhibited significant expression changes between G4 and G3. In the heatmap, each row represents an individual significant *O*-glycan. A red cell denotes a high relative abundance, while green represents a low relative abundance of the *O*-glycan.

The pie charts shown in Figure 3a,b depict the distribution changes in the glycan types of the 29 significant *O*-glycans from G4 and G3. The most abundant *O*-glycan in G3 was the neutral compositions (without fucoses and sialic acids), which took up 43.2%. However, this percentage was 18.9% lower than the percentage in G4, and 24.0% of the *O*-glycans in G3 were fucosylated, which is the same percentage as for the sialylated and fucosylated structures. The fucosylation was significantly increased in G3 compared with 4.8% in the control group (G4). Comparing the overall *O*-glycan distributions, an increased fucosylation was also observed in G3 when it was compared with G4, as shown in the Supplementary Materials Figure S2. This increased fucosylation level may be caused by proteinuria and hypertension progression, which merits additional attention in a future study. The sialylated *O*-glycans in G3 took up 8.9%, which was slightly lower than in the G4 control group. Figure 3c is a heatmap of differentially expressed *O*-glycans between G3 and G4. In the heatmap, each row represents the relative abundance of a significant *O*-glycan in two compared groups and each column is the replicate of each group. A high relative abundance was colored in red while low relative abundance appears in green. Among these 29 significant *O*-glycans, 11 of them had higher relative abundance in G3, with 3 sialylated, 2 fucosylated, 4 sialylated and fucosylated, and 2 others. On the other hand, 18 of them showed lower relative abundance in G3, including 3 sialylated, 2 fucosylated, 6 sialylated and fucosylated, and 7 others. The different *O*-glycan expressions that may be caused by proteinuria and hypertension progression should be further investigated in future studies.

For the female groups, 21 individual *O*-glycans were found to be statistically different in their expression between the two cohorts. As displayed in the Supplementary Materials Figure S4, the relative abundances of 21 *O*-glycans were significantly different in the expressions between G2 and G1. The distributions of different types of *O*-glycans were also compared between G1 and G2, as shown in Supplementary Materials Figure S5. The fucosylation level was 32.1% in G1, which was 27.9% higher than that in G2. When compared with the two controls (G2 and G4), higher abundance of fucosylated glycans from both the female and male disease-related groups (G1 and G3) could be associated with the higher activity of fucosyltransferases in the Golgi apparatus due to proteinuria and hypertension. Interestingly, neutral compositions took up 27.3% in G2 while this type was barely found in G1. When comparing the relative abundance of *O*-glycans between two healthy controls (G2 and G4), a total of 29 *O*-glycans were significantly different in the expressions between them, as shown in the Supplementary Materials Figure S6. Among these *O*-glycans, other *O*-glycans (without sialic acid and fucose) took up to 63% in both groups. Sialylated *O*-glycans took up 12.5% and 11.4% in G2 and G4, respectively. The number of fucosylated *O*-glycans was 6.3% higher while the number of sialylated and fucosylated *O*-glycans is 6.8% lower in G2 than in G4 (Supplementary Materials Figure S7). The different *O*-glycan expressions due to gender in the two healthy controls are consistent with the previous study [54].

### 3.4. Comparison between the Obese and Diabetic Group and the Control Group

**Figure 4:**
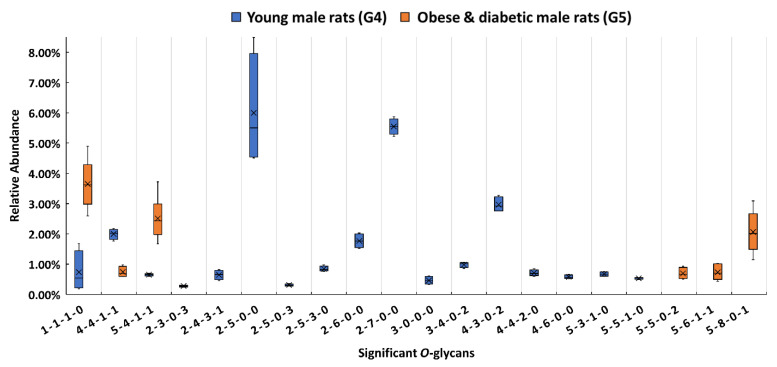
Box plot for relative abundance of significant *O*-glycans (*p* < 0.001) between male control (G4) and obese and diabetic male (G5). The putative structure was assigned to each glycan composition. The X axis denotes the four-digit codes for *O*-glycan compositions. The Y axis is the relative abundance. Error bars are the 95% confidence interval.

Along with the different glycan expressions in the group with proteinuria and hypertension, we also investigated the glycan expression changes that might be associated with obesity and diabetes. A LC–MS/MS analysis permitted the identification and quantification of 20 *O*-glycans, which had significant relative abundance changes between the obese and diabetic male group (G5) and the male control group (G4). According to the statistical *t*-test with Bonferroni correction, these *O*-glycans have a *p*-value less than 0.001, which illustrated statistically significant abundance changes between G5 and G4. The comparison of these 20 *O*-glycans is displayed as a box plot in Figure 4. For sialylated glycans, 5-5-0-2 only existed in G5, while 2-3-0-3, 2-5-0-3, and 3-4-0-2 were only detected in G4. For fucosylated *O*-glycans, 1-1-1-0 tended to increase from healthy controls (G4) to the obese and diabetic group (G5) while 2-5-3-0, 4-4-2-0, 5-3-1-0, and 5-5-1-0 were only expressed in G4. For *O*-glycans with both sialic acid and fucose, a lower level of expression was observed in 4-4-1-1 while an increased expression of 5-4-1-1 was shown in G5. For neutral compositions, 2-5-0-0, 2-6-0-0, 2-7-0-0, 3-0-0-0, and 4-6-0-0 were only found in G4, a similar result to the proteinuria and hypertension group. The expressions of all of these significant *O*-glycans were consistent with the MRM results, as shown in the Supplementary Materials Table S3.

**Figure 5:**
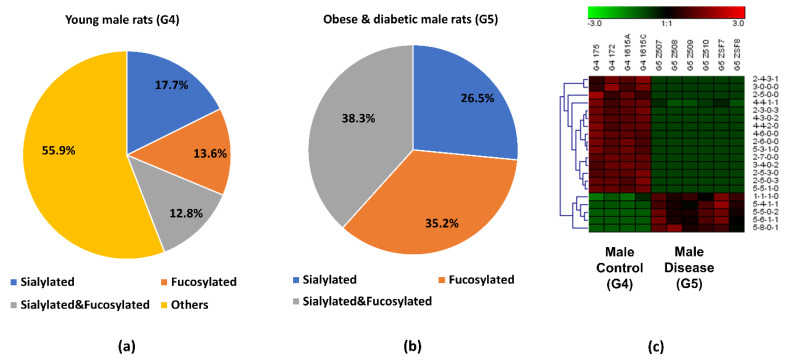
Distribution changes in the different *O*-glycan types from (**a**) the male control (G4) and (**b**) the obese and diabetic male (G5); (**c**) heatmap of 20 *O*-glycans that exhibited significant expression changes between G4 and G5. In the heatmap, each row represents an individual significant *O*-glycan. A red cell denotes a high relative abundance, while green represents a low relative abundance of the *O*-glycan.

Upon closer investigation of the distribution changes of different *O*-glycan types between G5 and G4, more detailed information was acquired for the 20 significant *O*-glycans. As shown in Figure 5a,b, sialylated *O*-glycans were 26.5% in G5, which was 8.8% higher than in G4. The percentage of the fucosylated *O*-glycans was 35.2% in G5 while it was only 13.6% in G4. When comparing the overall *O*-glycan distribution, the fucosylation level was more than two-fold higher in G5 (Supporting Information Figure S2)., The sialylated and fucosylated *O*-glycans in the obese and diabetic group took up 38.3% which was 25.5% higher than in the control. The increased percentage of this type of composition was also observed in the overall *O*-glycan distribution as shown in Supporting Information Figure S2. The sialylated and fucosylated compositions that contributed to this high percentage may be useful for diagnosis of obesity and diabetes. Interestingly, *O*-glycans without sialic acids and fucoses took up 55.9% in the control group. Figure 5c displays the heatmap of differentially expressed *O*-glycans between G4 and G5. When the relative abundances of the 20 significant *O*-glycans between the two groups were compared, 15 of them showed lower relative abundance in G5, with 4 sialylated, 4 fucosylated, 2 sialylated and fucosylated, and 5 others. Five out of 20 had higher relative abundance in G5, which included 2 sialylated, 1 fucosylated, and 2 sialylated and fucosylated *O*-glycans. The different *O*-glycan expressions and the role that *O*-glycans play in obese and diabetic progression is important and should be explored in future studies. It is envisaged that understanding the differences in the determinants of specific membrane glycosylation between obesity and diabetes could provide alternative approaches to disease diagnosis and ultimately lead to the development of tailored diabetes treatments.

## 4. Discussion

New analytical techniques that facilitate the discovery of potential glycan biomarkers are increasingly important in clinical diagnoses and therapies [55]. The use of *O*-glycans as potential biomarkers for cancer diagnostics has been reported in cancer cell lines, ovarian tissue, and blood serum studies [15,24,56]. However, *O*-glycomic studies on BBMs that are related to proteinuria, hypertension, obesity, and diabetes are seldom reported. To study the important role that *O*-glycans play in these chronic medical conditions, we performed *O*-glycomic profiling on BBMs of kidney cortices from rat models to investigate the different *O*-glycan expressions. To demonstrate the implementation of our *O*-glycomic profile method, a total of 22 BBM samples from five groups of rat kidney cortices were studied using LC–MS/MS analysis. The samples included four female and four male healthy controls, four females with proteinuria, four males with proteinuria and hypertension, and six males with obesity and diabetes. As the glycosylation of proteins in rats can vary by gender, the *O*-glycan expressions were compared within the same gender to avoid gender bias.

*N*-glycan profiling techniques have been successfully developed owing to the availability and high efficiency of a reliable enzyme for the release of *N*-glycans [57]. However, there are fewer efficient and reliable enzymes that can achieve *O*-glycan release. Given the lack of efficient enzymatic tools, chemical methods such as *β*-elimination are commonly used to release *O*-glycans [58,59,60], whereas these methods have some shortages including extra steps to remove excess salt, the cause of the peeling effect, a requirement of completely anhydrous conditions, and extreme caution with the use of toxic hydrazine. In the present study, we used an enzymatic/chemical approach that conducted *β*-elimination in milder reaction conditions [41]. This approach firstly used a nonspecific proteolysis for digestion followed by a solid-phase permethylation procedure for glycan derivatization. During the reaction, nonreduced *O*-glycans were immediately converted into permethylated glycans, which helped to reduce the peeling reaction. Permethylation helped to improve the ionization efficiency and to stabilize the sialic acids of *O*-glycans for positive electrospray ionization (ESI) [42,43]. It has also been reported that the permethylation of glycopeptides can only release *O*-glycans [41]. Moreover, this efficient and repeatable approach involved minimal sample handling and avoided the use of several hazardous chemicals. In the LC system, using a C18 trap for online purification to remove excess salt and impurities enabled better ionization of permethylated *O*-glycans. In the MS system, the LTQ Orbitrap Velos mass spectrometer offered high resolution, mass accuracy, and sensitivity, making the detection and identification of low abundant *O*-glycans possible. Due to the efficient and sensitive analysis of these techniques, a total of 59 *O*-glycans were identified and quantified. *O*-glycans that showed significant expressions were also confirmed with MRM LC–MS/MS experiments. Although their relative abundances were low, their S/N ratio were above 43 times higher than the limit of quantitation (S/N = 10). In this case, LC–MS/MS can detect all of these low abundant *O*-glycans and provided reliable data for quantification.

From the PCAs, clusters of five BBM groups are clearly separated apart, indicating significant *O*-glycan expression differences among these groups. *O*-glycans that showed significant expressions have larger contributions for the differentiation of BBM groups, resulting in the separation of the PCA clusters. Since the two PCA clusters of two healthy controls were separated, the difference is mainly due to gender, which further illustrates that it is necessary to compare the disease groups with the controls within the same gender. G3, G4, and G5 showed their clusters separated in the PCA plots, implying that their significant *O*-glycan expressions might be caused by the progression of proteinuria, hypertension, obesity, and diabetes.

Altered *O*-glycomes were observed between the sample groups with proteinuria and hypertension to the control groups. The fucosylation levels were higher in the female groups with mild proteinuria (G1) and the male group with sever proteinuria (G3) when they were compared with the two healthy controls. The altered fucosylation may be related to the different expressions of fucosyltransferases in Golgi cisternae between the disease-related and the control groups. However, due to the complex enzyme activity that contributed to the *O*-glycosylation modification, the conclusion that different expressions of *O*-glycans are related to specific enzymes needs further study. Comparing the overall *O*-glycan distributions between two controls (Supplementary Materials Figure S2), the distributions of sialylated *O*-glycans in G2 was higher while the expression of neutral *O*-glycans in G2 was lower than that in G4, which is mainly due to the gender difference. The increased fucosylation level in G3 is associated with proteinuria and hypertension progression. However, whether symptoms such as proteinuria and hypertension are due to altered fucosylation or the altered fucosylation was due to symptoms that merit additional investigation in a future study. Interestingly, when the male disease group (G3) and the female disease group (G1) were compared with the corresponding healthy controls, we found that 3-1-0-4 was expressed only in the two disease groups. The expression of this *O*-glycan may be related to the proteinuria. However, the expressions of 2-7-0-0, 4-5-0-0, and 4-3-2-1 existed only in the two controls. This result suggests that proteinuria progression may inhibit the formation of these *O*-glycans. The expression trend of neutral *O*-glycans between G2 and G1 was consistent with the comparison result between G3 and G4, while other glycans were expressed differently between the two group comparisons. This result is expected since glycosylation has been reported to differ between genders [54]. Moreover, proteinuria in the female group (G1) was less severe than in the male group (G3), which may have caused the differing glycan expressions.

*O*-glycan alterations that were associated with diabetes have been observed in the diabetic ovarian tissues of mouse and gastric cancer patients compared with in the healthy controls [24,25]. Fewer *O*-glycans were observed in the obese and diabetic groups compared with the controls. For example, *O*-glycans without sialic acids and fucoses were barely found in the obese and diabetic group. It has been reported that the process of the Mucin type glycosylation formation is catalyzed by *N*-acetylgalactosaminyl transferases (GalNAc-transferases) from a family of uridine diphosphate-*N*-acetylgalactosamine [61]. A lack of expression or low expression of certain *O*-glycans may be related to the activity of GalNAc-transferases, which could possibly be influenced by the obese and diabetic symptoms. The fucosylation level was more than two-fold higher in G5, indicating that the progression of obese and diabetic symptoms may contribute to a change in *O*-glycan fucosylation. Moreover, the sialylated and fucosylated compositions that contributed to a high percentage (38.3%), which is 25.5% higher than the control, may be useful for a diagnosis of obesity and diabetes. In the previous study, it was reported that diabetes exhibits major metabolic alternation, which affects and alters *O*-GlcNAcylation [62]. The altered expression of *O*-glycans may be associated with the aberrant glycosylation caused by obese and diabetic progression.

## 5. Summary

In this study, we performed a comprehensive glycomic profiling of five groups of BBM samples to study *O*-glycan expressions in CKD-related symptoms including proteinuria, hypertension, obesity, and diabetes. Groups with proteinuria, hypertension, obesity, and diabetes exhibited distinguishable *O*-glycan expressions compared with the two control groups. The fucosylation level was higher in both G1 and G3 when compared with two respective controls, indicating that glycan fucosylation may be associated with proteinuria. The altered fucosylation may be related to the different expressions of fucosyltransferases in Golgi cisternae caused by proteinuria. Increased relative abundances of *O*-glycans 1-1-1-0, 5-4-1-0, 2-4-1-2, and 2-5-2-1 were observed in G3, indicating that these *O*-glycans may be tightly connected with the progression of proteinuria and hypertension. A lower level of expression was observed with 4-4-1-1, while increased expressions of 1-1-1-0 and 5-4-1-1 were observed in G5, suggesting that the change in expressions from these *O*-glycans may contribute to obesity and diabetes. The altered expressions of these O-glycans may be used as potential indicators for CKD. The alternation of sialylation and fucosylation that existed in the disease groups and control groups suggested an association with proteinuria, hypertension, obesity, and diabetes. These glycomic results provide important clues for better understanding the *O*-glycan expressions in CKD progressions that include proteinuria, hypertension, obesity, and diabetes and may facilitate the discovery of *O*-glycans as promising indicators for early diagnosis and improved treatment of CKD.

## Figures and Tables

**Table 1:** Different specifications of the five groups of rats.

Group	G1	G2	G3	G4	G5
Rat Strain	Munich Wistar Frömter (MWF)	MWF	MWF	MWF	Zucker SF1 (ZSF1) obese
Gender	Female	Female	Male	Male	Male
Age–weeks	32–42	<10	32–42	7	16
Physiology	Mild proteinuria	Normal	Proteinuria & mild hypertension	Normal	Obese, hypertension, hyperlipidemia, hyperglycemia
Urinary protein (mg/day)	<100	Negligible	>400	<50	~400

## Data Availability

The research data used in this article are available from the corresponding author upon request.

## Supplementary Materials

The following are available online at https://www.mdpi.com/article/10.3390/biom11111560/s1, Figure S1: An example of *O*-glycan (1-1-1-2) identification with full MS and MS^2^; Figure S2: Distribution of the different types of *O*-glycans among five groups; Figure S3: The PCA plots for (**a**) the young female control group (G2) and the young male control group (G4), and (**b**) the older female group with proteinuria (G1) and the young female control group (G2); Figure S4: Box plot for relative abundance of 21 significant *O*-glycans (*p* < 0.001) between the young female control group (G2) and older female group with proteinuria (G1); Figure S5: Distribution of the types of *O*-glycans and a heatmap of significant *O*-glycans from the young female control group (G2) and the older female group with proteinuria (G1); Figure S6: Box plot for relative abundance of significantly expressed *O*-glycans (*p* < 0.001) between the young female control group (G2) and the young male control group (G4); Figure S7: Distribution of the types of *O*-glycans and a heatmap of significant *O*-glycans from the young female control group (G2) and the young male control group (G4); Table S1: List of all identified *O*-glycans with their theoretical *m*/*z*, observed *m*/*z*, average relative abundance, standard deviation, and *p*-value for each sample group. This table is presented in an attached Excel file; Table S2: This table is presented in an attached Excel file that contains a list of statistically significant *O*-glycans (*p* < 0.001) between the older male group with proteinuria and hypertension (G3) versus the male control group (G4), the obese and diabetic male group (G5) versus the male control group (G4), the older female group with proteinuria (G1) versus the female control group (G2), and the female control group (G2) versus the male control group (G4) and their relative abundance with *p*-value; Table S3: Transitions used for the quantitation of permethylated *O*-glycans detected in five BBM groups for MRM LC–MS/MS.
