# Supplementary material for: Altered O-glycomes of Renal Brush-Border Membrane in Model Rats with Chronic Kidney Diseases
Source: Biomolecules. 2021 Oct 21;11(11):1560. doi: 10.3390/biom11111560 (PMC8615448; doi:10.3390/biom11111560)
Supplement: Supplementary file 1 [file biomolecules-11-01560-s001.zip › Manuscript_BBM O glycan_Supporting information Revision 2 .pdf]

Supporting Information

Altered *O*-Glycomes of Renal Brush-border Membrane in Model Rats with Chronic Kidney Diseases

Aiying Yu[1], Jingfu Zhao[1], Jieqiang Zhong[1], Junyao Wang[1], Shiv Pratap S. Yadav[2], Bruce A Molitoris[2], Mark C Wagner[2], and Yehia Mechref[1*]

[1]Department of Chemistry and Biochemistry, Texas Tech University, Texas, TX, 79409, United States; aiying.yu@ttu.edu (A.Y.); jingfu.zhao@ttu.edu (J.Z.); jieqiang.zhong@ttu.edu (J.Z.); junyao.wang@ttu.edu (J.W.).
[2]Nephrology Division, Department of Medicine, Indiana University, Indianapolis, IN, 46202, United States; ssyadav@iu.edu (S.P.S.Y.); bmolitor@iu.edu (B.A.M.); wagnerm@iu.edu (M.C.W.).
*Correspondence: yehia.mechref@ttu.edu (Y.M.); Tel: 806-742-3059
Keywords: *O*-glycan, Brush-border membrane, Proteinuria and hypertension, Obese and diabetic, Chronic kidney disease, Differential expression analysis, LC-MS/MS

Table of Contents

Supporting Information Table S1. List of all identified *O*-glycans with their theoretical *m/z*, observed *m/z*, average relative abundance, standard deviation, and *p*-value for each sample group. This table is presented in an attached Excel file.

Supporting Information Table S2. This table is presented in an attached Excel file which contains a list of statistically significant *O*-glycans (p<0.001) between old male group with proteinuria and hypertension (G3) *versus* male control group (G4), Obese and diabetic male group (G5) *versus* male control group (G4), old female group with proteinuria (G1) *versus* female control group (G2), and female control group (G2) *versus* male control group (G4) and their relative abundance with *p*-value.

Supporting Information Table S3. Transitions used for the quantitation of permethylated *O*-glycans detected in five BBM groups for MRM LC-MS/MS.

Supporting Information Figure S1. An example of identification of *O*-glycan ($HexNAc_1Hex_1NeuAc_2$) with full MS and MS^2.

Supporting Information Figure S2. Distribution of the different types of *O*-glycans among five groups.

Supporting Information Figure S3. The PCA plots for **(a)** the young female control group (G2) and the young male control group (G4), and **(b)** the old female group with proteinuria (G1) and the young female control group (G2).

Supporting Information Figure S4. Box plot for relative abundance of 21 significant *O*-glycans ($p<0.001$) between the young female control group (G2) and old female group with proteinuria (G1).

Supporting Information Figure S5. Distribution of the types of *O*-glycans and a heatmap of significant *O*-glycans from the young female control group (G2) and old female group with proteinuria (G1).

Supporting Information Figure S6. Box plot for relative abundance of significantly expressed *O*-glycans ($p<0.001$) between the young female control group (G2) and the young male control group (G4).

Supporting Information Figure S7. Distribution of the types of *O*-glycans and a heatmap of significant *O*-glycans from the young female control group (G2) and the young male control group (G4).

Table S3. Transitions used for the quantitation of permethylated *O*-glycans detected in five BBM groups for MRM LC-MS/MS. Symbols: see **Figure S1.**

Structures	*m/z* (charge)	Transitions			Collision Energy
1-1-0-1 *	857.450 (+1)	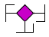 BZZ 312	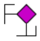 BZ 344	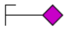 B 376	35
1-1-1-0 #	670.365 (+1)	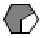 ${}^{0,2}\mathrm{X}$ 111	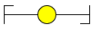 BZ 187	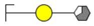 $\mathrm{B}^{0,2}\mathrm{X}$ 262	35
2-0-0-0 *	559.284 (+1)	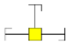 BZZ 196	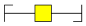 BZ 228	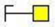 B 260	30
2-0-1-0	711.392 (+1)	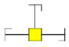 BZZ 196	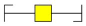 BZ 228	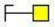 B 260	35
2-1-0-0	741.402 (+1)	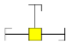 BZZ 196	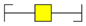 BZ 228	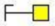 B 260	35
2-10-0-0	1289.654 (+2)	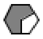 ${}^{0,2}\mathrm{X}$ 111	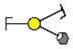 $\mathrm{BZ}^{0,2}\mathrm{X}$ 230	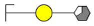 $\mathrm{B}^{0,2}\mathrm{X}$ 262	35
2-1-0-1	1102.576 (+1)	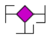 BZZ 312	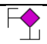 BZ 344	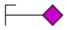 B 376	30

2-1-0-2	1463.750<br>(+1)	BZZ<br>312	BZ<br>344	B<br>376	35
2-3-0-0 *	1171.584<br>(+1)	$^{0,2}X$<br>111	$BZ^{0,2}X$<br>230	$B^{0,2}X$<br>262	35
2-3-0-3 [#]	1125.579<br>(+2)	BZZ<br>312	BZ<br>344	B<br>376	35
2-3-1-2 *	1040.550<br>(+2)	BZZ<br>312	BZ<br>344	B<br>376	35
2-4-1-2 *	1142.600<br>(+2)	BZZ<br>312	BZ<br>344	B<br>376	35
2-4-2-1 *	1040.544<br>(+2)	BZZ<br>312	BZ<br>344	B<br>376	35
2-4-2-2	1212.618<br>(+2)	BZZ<br>312	BZ<br>344	B<br>376	35
2-4-3-1 *	1127.589<br>(+2)	BZZ<br>312	BZ<br>344	B<br>376	35
2-5-0-0 * [#]	787.918<br>(+2)	BZ<br>187	$B^{0,2}X$<br>262	$C^{2,5}X$<br>294	35
2-5-0-3 * [#]	1338.192<br>(+2)	BZZ<br>312	BZ<br>344	B<br>376	35
2-5-1-3	1416.723<br>(+2)	BZZ<br>312	BZ<br>344	B<br>376	35
2-5-2-1 *	1134.081<br>(+2)	BZZ<br>312	BZ<br>344	B<br>376	35
2-5-3-0 * [#]	1040.538<br>(+2)	BZ<br>187	$B^{0,2}X$<br>262	$C^{2,5}X$<br>294	35
2-5-3-2	940.481<br>(+3)	BZZ<br>312	BZ<br>344	B<br>376	35
2-6-0-0 * [#]	889.968<br>(+2)	$^{0,2}X$<br>111	$BZ^{0,2}X$<br>230	$B^{0,2}X$<br>262	35
2-7-0-0 * [#]	992.018<br>(+2)	$^{0,2}X$<br>111	$BZ^{0,2}X$<br>230	$B^{0,2}X$<br>262	35
3-0-0-0 [#]	799.456<br>(+1)	BZZ<br>196	BZ<br>228	B<br>260	35
3-1-0-4 *	1216.116<br>(+2)	BZZ<br>312	BZ<br>344	B<br>376	35

3-2-1-0	1381.744 (+1)	BZ 187	BZZ 196	BZ 228	45
3-2-2-0	769.907 (+2)	BZ 187	BZZ 196	BZ 228	45
3-4-0-2 #	1178.118 (+2)	BZZ 312	BZ 344	B 376	35
3-5-1-1	1178.113 (+2)	BZZ 312	BZ 344	B 376	35
3-6-0-1 *	1184.605 (+2)	BZZ 312	BZ 344	B 376	35
3-6-1-1	1271.649 (+2)	BZZ 312	BZ 344	B 376	35
4-3-0-2 *	1198.632 (+2)	BZZ 312	BZ 344	B 376	35
4-3-2-1 *	1192.134 (+2)	BZZ 312	BZ 344	B 376	35
4-4-1-1 * #	1198.626 (+2)	BZZ 312	BZ 344	B 376	35
4-4-2-0 #	1096.570 (+2)	BZ 187	BZ 228	BZ 432	35
4-5-0-0 *	1024.531 (+2)	BZ 187	BZ 228	BZ 432	35
4-5-1-1	1300.676 (+2)	BZZ 312	BZ 344	B 376	35
4-5-1-2 *	987.842 (+3)	BZZ 312	BZ 344	B 376	35
4-6-0-0 * #	1126.581 (+2)	BZ 187	$B^{0,2}X$ 262	BZ 432	35
5-3-1-0 #	1030.039 (+2)	BZZ 196	BZ 228	B 260	35
5-4-0-0	1045.044 (+2)	BZZ 196	BZ 228	B 260	35
5-4-1-0 *	1132.089 (+2)	BZ 228	B 260	B 464	35
5-4-1-1 #	1312.676 (+2)	BZZ 312	BZ 344	B 376	35

5-5-0-0 *	1147.094 (+2)	BZZ 196	BZ 228	BZ 432	35
5-5-0-2 * #	1011.526 (+3)	BZZ 312	BZ 344	B 376	35
5-5-1-0 #	1234.139 (+2)	BZZ 196	BZ 228	BZ 432	35
5-6-1-1 *	1011.520 (+3)	BZZ 312	BZ 344	B 376	35
5-8-0-1	1106.578 (+3)	BZZ 312	BZ 344	B 376	35
5-8-1-0 *	1038.544 (+3)	BZZ 196	BZ 228	BZ 432	35

*Significant *O*-glycans ($p<0.05$) between old male group with proteinuria and hypertension (G3) and young male control group (G4) were confirmed from MRM experiment.

#Significant *O*-glycans ($p<0.05$) between obese and diabetic male group (G5) and young male control group (G4) were confirmed from MRM experiment.

Supporting Information Figure S1

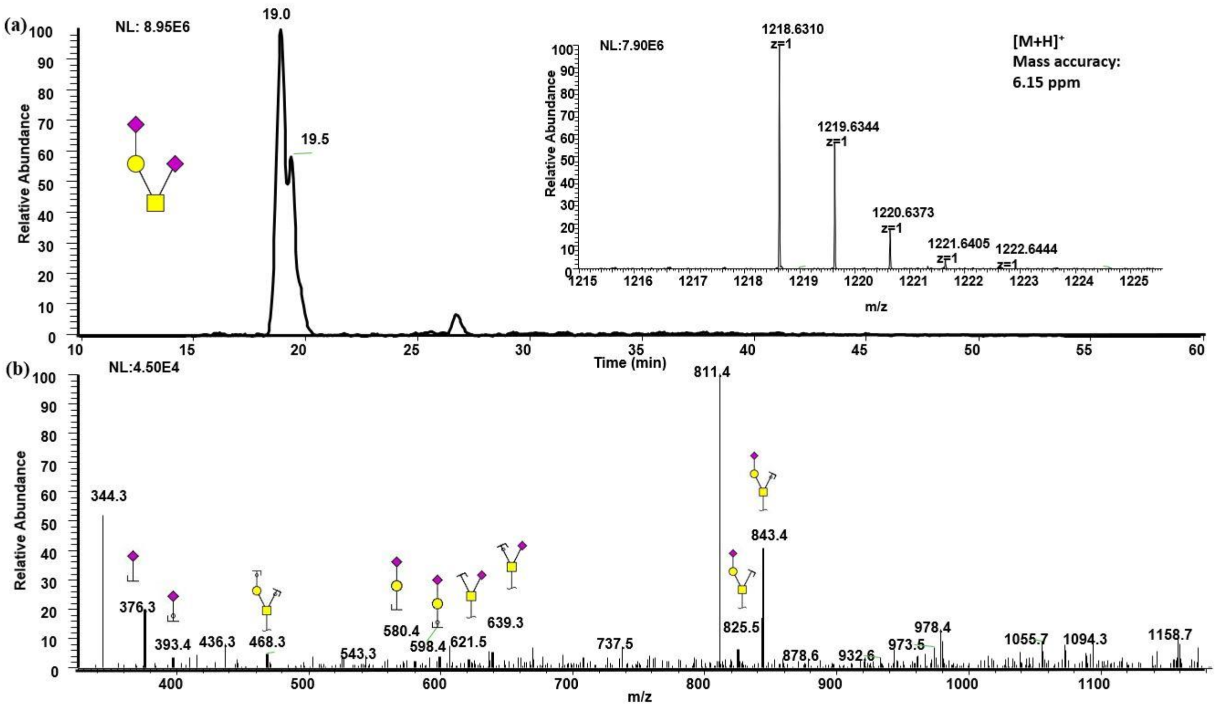

Supporting Information Figure S1. An example of *O*-glycan identification with full MS and MS^2. **(a)** The EIC of *O*-glycan $HexNAc_1Hex_1NeuAc_2$, inset is the full MS. **(b)** MS^2 for $HexNAc_1Hex_1NeuAc_2$. HexNAc includes *N*-acetylglucosamine and *N*-acetylgalactosamine. Hexose includes galactose, glucose, and mannose. Deoxyhexose is fucose and NeuAc is *N*-acetylneuraminic acid. Symbols: ,*N*-acetylgalactosamine; ,Galactose (Gal); , *N*-acetylneuraminic acid (NeuAc/Sialic Acid).

Supporting Information Figure S2

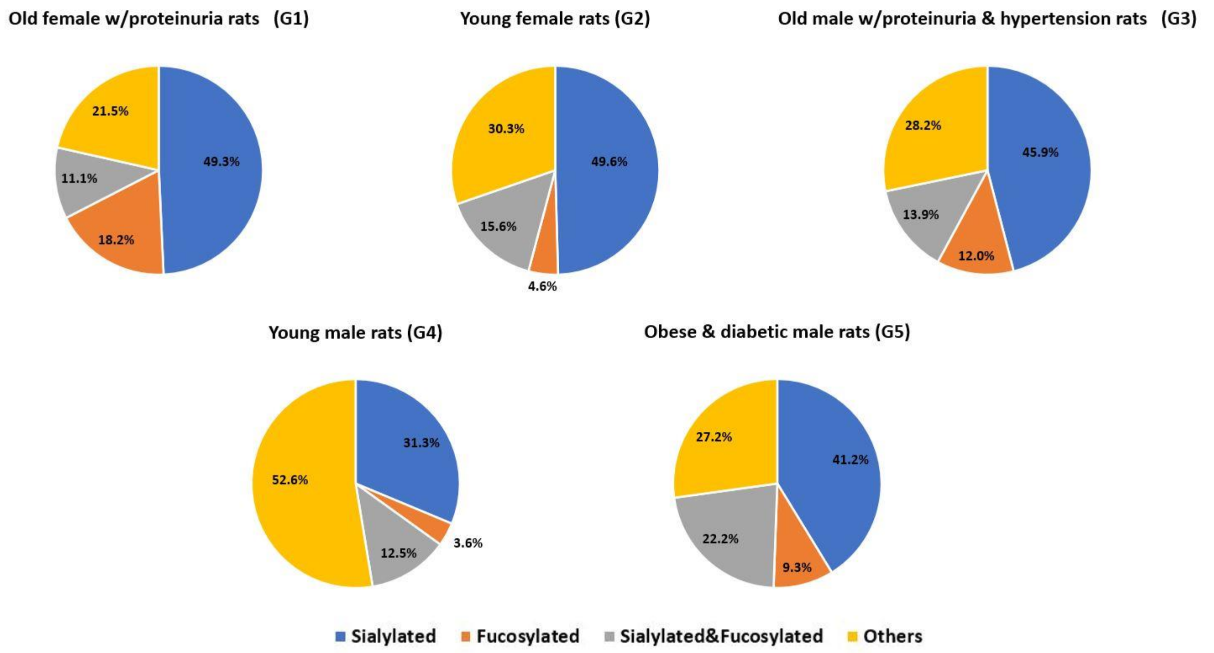

Supporting Information Figure S2. Distribution of the different *O*-glycans types among five groups.

Supporting Information Figure S3

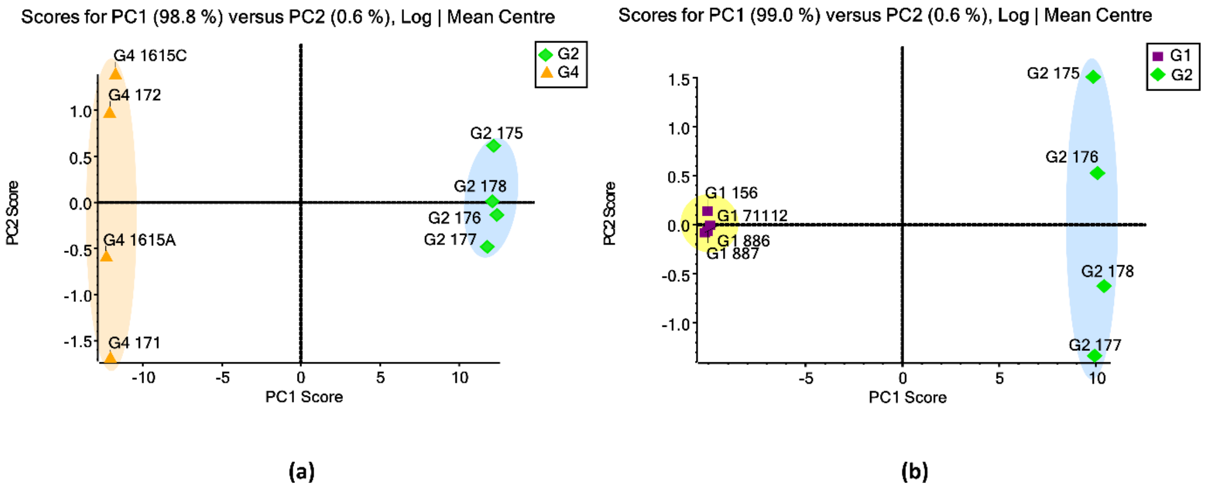

Supporting Information Figure S3. The PCA plots for **(a)** the young female control group (G2) and the young male control group (G4), and **(b)** old female group with proteinuria (G1) and the young female control group (G2).

Supporting Information Figure S4

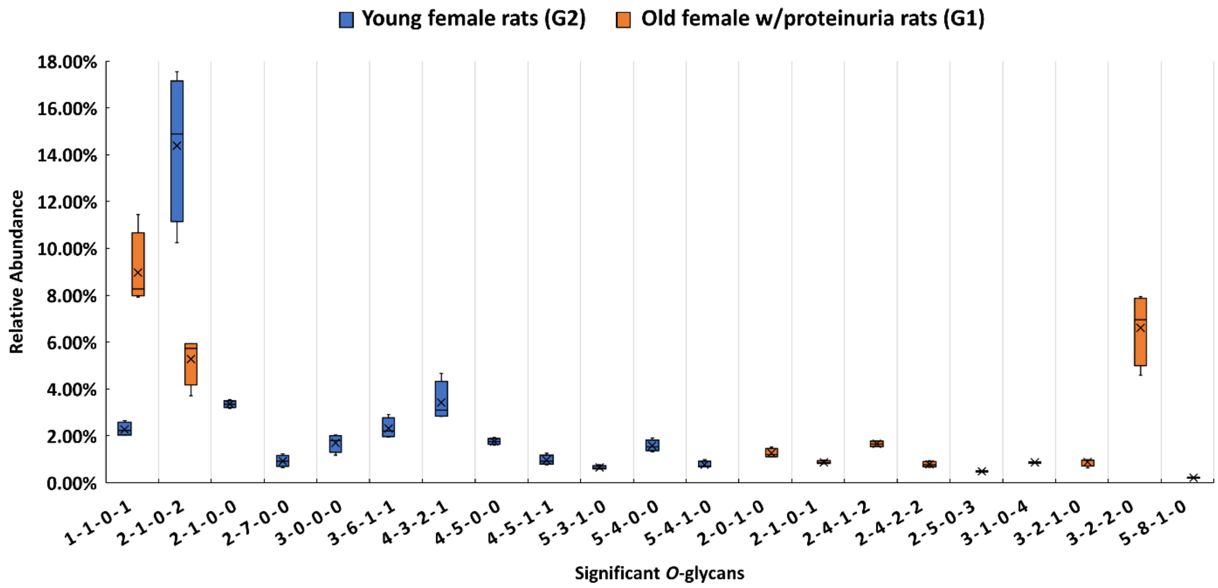

Supporting Information Figure S4. Box plot for relative abundance of 21 significant *O*-glycans (p<0.001) between the young female control group (G2) and old female group with proteinuria (G1). The four-digit codes represent *O*-glycan compositions. Error bars are the 95% confidence interval. X-X-X-X stands for HexNAc-Hexose-DeoxyHex-NeuAc. HexNAc

includes N-acetylglucosamine and N-acetylgalactosamine. Hexose includes galactose, glucose, and mannose. Deoxyhexose is fucose and NeuAc is N-acetylneuraminic acid.

Supporting Information Figure S5

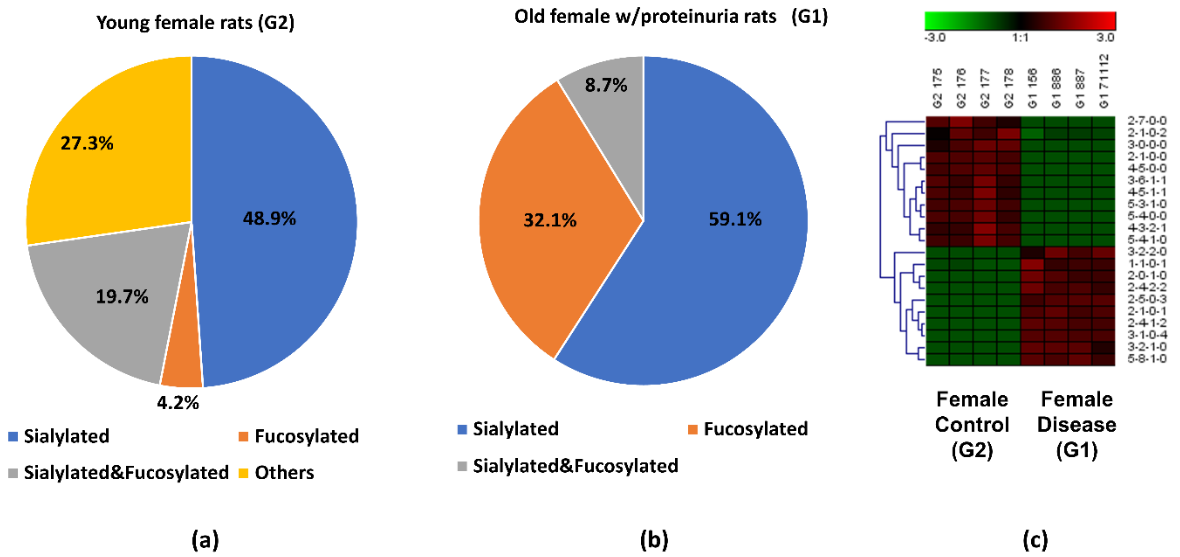

Supporting Information Figure S5. Distribution of the types of *O*-glycans derived from **(a)** the young female control group (G2) and **(b)** the old female group with proteinuria (G1); **(c)** heatmap of 21 *O*-glycans that exhibited significant expression changes between G2 and G1.

Supporting Information Figure S6

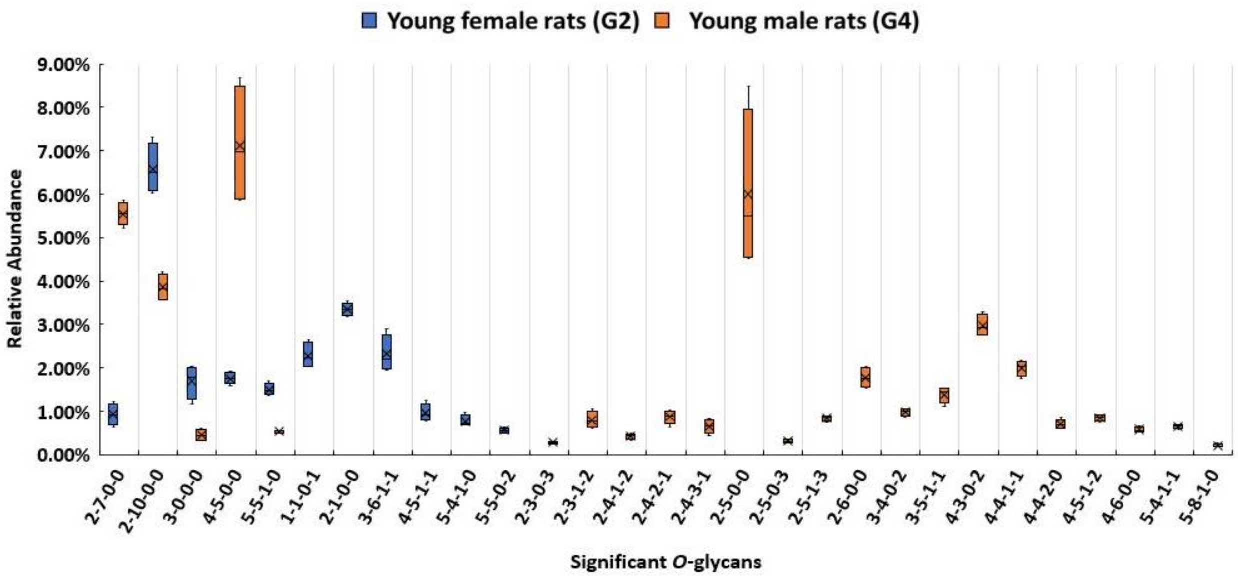

Supporting Information Figure S6. Box plot for relative abundance of significantly expressed *O*-glycans (p<0.001) between the female control group (G2) and the male control group (G4). Error bars are the 95% confidence interval.

Supporting Information Figure S7

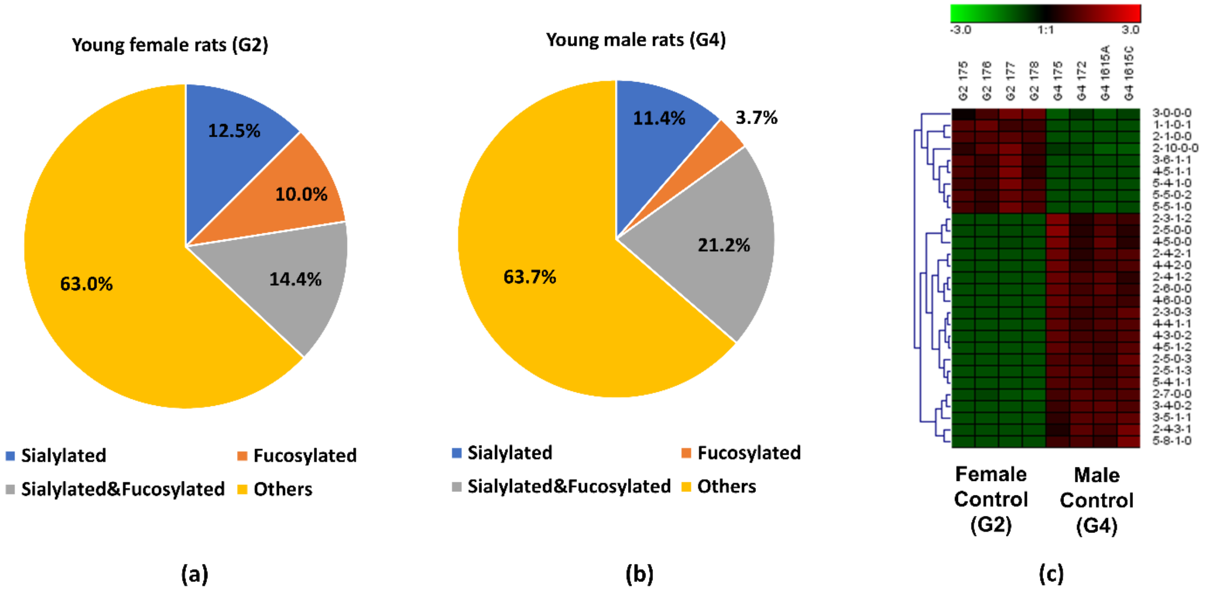

Supporting Information Figure S7. Distribution of the types of *O*-glycans derived from **(a)** the female control group (G2) and **(b)** the male control group (G4); **(c)** heatmap of 29 *O*-glycans that exhibited significant expression changes between G2 and G4.
