# Supplementary material for: Altered O-glycomes of Renal Brush-Border Membrane in Model Rats with Chronic Kidney Diseases
Source: Biomolecules. 2021 Oct 21;11(11):1560. doi: 10.3390/biom11111560 (PMC8615448; doi:10.3390/biom11111560)
Supplement: Supplementary file 1 [file biomolecules-11-01560-s001.zip › Statistically significant O-glycan G3G4 G4G5 G1G2 G2G4 BioM.pdf]

Table S2. List of Statistically significant O-Glycans in old male group with proteinuria and hypertension (G3) and young male control group (G4), male group with obesity and diebetics (G5) and male control group (G4), old female group with proteinuria (G1) and young female control group (G2), and young female control group (G2) and young male control group (G4) and their relative abundance with p-value.

The four-digit codes represent *O*-glycan compositions. X-X-X-X stands for HexNAc-Hexose-DeoxyHex-NeuAc. HexNAc includes N-acetylglucosamine and N-acetylgalactosamine. Hexose includes galactose, glucose, and mannose. Deoxyhexose is fucose and NeuAc is N-acetylneuraminic acid.

**Statistically significant *O*-Glycans in old male group with proteinuria and hypertension (G3) and young male control group (G4)**													
Glycan	G3 44A	G3 44B	G3 45A	G3 45C	Average relative abundance	Standard deviation	G4 175	G4 172	G4 1615A	G4 1615C	Average relative abundance	Standard deviation	p-value(Bonferroni correction)
1-1-1-0	5.80E-02	5.48E-02	8.04E-02	5.11E-02	6.11E-02	1.32E-02	1.80E-03	7.55E-03	3.27E-03	1.68E-02	7.35E-03	6.73E-03	0.0003
2-0-0-0	9.28E-02	9.64E-02	1.39E-01	8.00E-02	1.02E-01	2.57E-02	0.00E+00	0.00E+00	0.00E+00	0.00E+00	0.00E+00	0.00E+00	0.0002
2-3-0-0	1.51E-02	8.28E-03	1.37E-02	1.75E-02	1.36E-02	3.90E-03	0.00E+00	0.00E+00	0.00E+00	0.00E+00	0.00E+00	0.00E+00	0.0004
2-3-0-3	0.00E+00	0.00E+00	0.00E+00	0.00E+00	0.00E+00	0.00E+00	2.95E-03	2.37E-03	2.55E-03	3.11E-03	2.75E-03	3.44E-04	0.000004
2-3-1-2	0.00E+00	0.00E+00	0.00E+00	0.00E+00	0.00E+00	0.00E+00	1.06E-02	6.03E-03	8.08E-03	7.22E-03	7.99E-03	1.95E-03	0.0002
2-4-1-2	2.33E-02	3.48E-02	2.87E-02	2.65E-02	2.83E-02	4.82E-03	4.91E-03	4.15E-03	4.53E-03	3.35E-03	4.23E-03	6.69E-04	0.00006
2-4-2-1	0.00E+00	0.00E+00	0.00E+00	0.00E+00	0.00E+00	0.00E+00	1.03E-02	6.45E-03	8.83E-03	8.97E-03	8.64E-03	1.60E-03	0.00004
2-4-3-1	0.00E+00	0.00E+00	0.00E+00	0.00E+00	0.00E+00	0.00E+00	4.45E-03	7.08E-03	6.07E-03	8.19E-03	6.45E-03	1.59E-03	0.0002
2-5-0-0	0.00E+00	0.00E+00	0.00E+00	0.00E+00	0.00E+00	0.00E+00	8.49E-02	4.51E-02	6.37E-02	4.63E-02	6.00E-02	1.86E-02	0.0007
2-5-0-3	0.00E+00	0.00E+00	0.00E+00	0.00E+00	0.00E+00	0.00E+00	3.11E-03	3.12E-03	2.66E-03	3.63E-03	3.13E-03	3.99E-04	0.000004
2-5-2-1	2.19E-02	3.04E-02	2.64E-02	2.63E-02	2.63E-02	3.45E-03	5.80E-03	5.57E-03	4.42E-03	6.15E-03	5.48E-03	7.48E-04	0.00002
2-5-3-0	0.00E+00	0.00E+00	0.00E+00	0.00E+00	0.00E+00	0.00E+00	7.55E-03	8.23E-03	8.20E-03	9.75E-03	8.43E-03	9.33E-04	0.000002
2-5-3-2	4.43E-03	5.32E-03	6.18E-03	4.73E-03	5.17E-03	7.72E-04	0.00E+00	0.00E+00	0.00E+00	0.00E+00	0.00E+00	0.00E+00	0.00001
2-6-0-0	0.00E+00	0.00E+00	0.00E+00	0.00E+00	0.00E+00	0.00E+00	2.03E-02	1.62E-02	1.90E-02	1.52E-02	1.77E-02	2.38E-03	0.000006
2-7-0-0	0.00E+00	0.00E+00	0.00E+00	0.00E+00	0.00E+00	0.00E+00	5.22E-02	5.87E-02	5.52E-02	5.58E-02	5.55E-02	2.65E-03	0.00000001
3-0-0-0	0.00E+00	0.00E+00	0.00E+00	0.00E+00	0.00E+00	0.00E+00	3.31E-03	6.10E-03	3.77E-03	5.28E-03	4.62E-03	1.30E-03	0.0004
3-1-0-4	9.56E-03	8.50E-03	1.14E-02	9.02E-03	9.61E-03	1.24E-03	0.00E+00	0.00E+00	0.00E+00	0.00E+00	0.00E+00	0.00E+00	0.000005
3-6-0-1	8.43E-03	1.08E-02	1.15E-02	9.99E-03	1.02E-02	1.32E-03	0.00E+00	0.00E+00	0.00E+00	0.00E+00	0.00E+00	0.00E+00	0.000005
4-3-0-2	0.00E+00	0.00E+00	0.00E+00	0.00E+00	0.00E+00	0.00E+00	3.28E-02	2.77E-02	2.76E-02	3.07E-02	2.97E-02	2.54E-03	0.0000004
4-3-2-1	0.00E+00	0.00E+00	0.00E+00	0.00E+00	0.00E+00	0.00E+00	2.76E-02	2.58E-02	2.43E-02	2.85E-02	2.66E-02	1.86E-03	0.0000001
4-4-1-1	0.00E+00	0.00E+00	0.00E+00	0.00E+00	0.00E+00	0.00E+00	2.18E-02	1.76E-02	1.99E-02	2.06E-02	2.00E-02	1.73E-03	0.0000004
4-5-0-0	0.00E+00	0.00E+00	0.00E+00	0.00E+00	0.00E+00	0.00E+00	8.67E-02	6.00E-02	7.94E-02	5.86E-02	7.12E-02	1.41E-02	0.00005
4-5-1-2	0.00E+00	0.00E+00	0.00E+00	0.00E+00	0.00E+00	0.00E+00	9.23E-03	8.06E-03	7.64E-03	8.88E-03	8.45E-03	7.28E-04	0.0000004
4-6-0-0	0.00E+00	0.00E+00	0.00E+00	0.00E+00	0.00E+00	0.00E+00	6.74E-03	5.70E-03	5.58E-03	5.23E-03	5.81E-03	6.46E-04	0.000002
5-4-1-0	2.46E-03	3.10E-03	3.52E-03	4.10E-03	3.30E-03	6.90E-04	0.00E+00	0.00E+00	0.00E+00	0.00E+00	0.00E+00	0.00E+00	0.00008
5-5-0-0	0.00E+00	0.00E+00	0.00E+00	0.00E+00	0.00E+00	0.00E+00	2.21E-02	1.39E-02	1.69E-02	1.42E-02	1.67E-02	3.80E-03	0.0001
5-5-0-2	3.48E-03	3.89E-03	4.72E-03	3.97E-03	4.01E-03	5.16E-04	0.00E+00	0.00E+00	0.00E+00	0.00E+00	0.00E+00	0.00E+00	0.000004
5-6-1-1	3.99E-03	3.83E-03	5.46E-03	4.59E-03	4.47E-03	7.39E-04	0.00E+00	0.00E+00	0.00E+00	0.00E+00	0.00E+00	0.00E+00	0.00002
5-8-1-0	0.00E+00	0.00E+00	0.00E+00	0.00E+00	0.00E+00	0.00E+00	1.94E-03	2.11E-03	1.76E-03	2.75E-03	2.14E-03	4.30E-04	0.00006

**Statistically significant *O*-Glycans in male group with obesity and diebetics (G5) and young male control group (G4)**															
Glycan	G4 175	G4 172	G4 1615A	G4 1615C	Average relative abundance	Standard deviation	G5 Z507	G5 Z508	G5 Z509	G5 Z510	G5 ZSF7	G5 ZSF8	Average relative abundance	Standard deviation	p-value(Bonferroni correction)
1-1-1-0	1.80E-03	7.55E-03	3.27E-03	1.68E-02	7.35E-03	6.73E-03	4.08E-02	3.11E-02	3.60E-02	2.59E-02	4.90E-02	3.63E-02	3.65E-02	7.96E-03	0.0003
2-3-0-3	2.95E-03	2.37E-03	2.55E-03	3.11E-03	2.75E-03	3.44E-04	0.00E+00	0.00E+00	0.00E+00	0.00E+00	0.00E+00	0.00E+00	0.00E+00	0.00E+00	0.00000004
2-4-3-1	4.45E-03	7.08E-03	6.07E-03	8.19E-03	6.45E-03	1.59E-03	0.00E+00	0.00E+00	0.00E+00	0.00E+00	0.00E+00	0.00E+00	0.00E+00	0.00E+00	0.000007
2-5-0-0	8.49E-02	4.51E-02	6.37E-02	4.63E-02	6.00E-02	1.86E-02	0.00E+00	0.00E+00	0.00E+00	0.00E+00	0.00E+00	0.00E+00	0.00E+00	0.00E+00	0.00004
2-5-0-3	3.11E-03	3.12E-03	2.66E-03	3.63E-03	3.13E-03	3.99E-04	0.00E+00	0.00E+00	0.00E+00	0.00E+00	0.00E+00	0.00E+00	0.00E+00	0.00E+00	0.00000004
2-5-3-0	7.55E-03	8.23E-03	8.20E-03	9.75E-03	8.43E-03	9.33E-04	0.00E+00	0.00E+00	0.00E+00	0.00E+00	0.00E+00	0.00E+00	0.00E+00	0.00E+00	0.00000001
2-6-0-0	2.03E-02	1.62E-02	1.90E-02	1.52E-02	1.77E-02	2.38E-03	0.00E+00	0.00E+00	0.00E+00	0.00E+00	0.00E+00	0.00E+00	0.00E+00	0.00E+00	0.00000007
2-7-0-0	5.22E-02	5.87E-02	5.52E-02	5.58E-02	5.55E-02	2.65E-03	0.00E+00	0.00E+00	0.00E+00	0.00E+00	0.00E+00	0.00E+00	0.00E+00	0.00E+00	0.00000000001
3-0-0-0	3.31E-03	6.10E-03	3.77E-03	5.28E-03	4.62E-03	1.30E-03	0.00E+00	0.00E+00	0.00E+00	0.00E+00	0.00E+00	0.00E+00	0.00E+00	0.00E+00	0.00002
3-4-0-2	8.62E-03	1.04E-02	1.06E-02	9.80E-03	9.86E-03	9.02E-04	0.00E+00	0.00E+00	0.00E+00	0.00E+00	0.00E+00	0.00E+00	0.00E+00	0.00E+00	0.000000003
4-3-0-2	3.28E-02	2.77E-02	2.76E-02	3.07E-02	2.97E-02	2.54E-03	0.00E+00	0.00E+00	0.00E+00	0.00E+00	0.00E+00	0.00E+00	0.00E+00	0.00E+00	0.000000002
4-4-1-1	2.18E-02	1.76E-02	1.99E-02	2.06E-02	2.00E-02	1.73E-03	9.06E-03	5.85E-03	5.99E-03	7.79E-03	9.83E-03	5.89E-03	7.40E-03	1.76E-03	0.000004
4-4-2-0	8.47E-03	5.96E-03	6.78E-03	7.07E-03	7.07E-03	1.05E-03	0.00E+00	0.00E+00	0.00E+00	0.00E+00	0.00E+00	0.00E+00	0.00E+00	0.00E+00	0.0000001
4-6-0-0	6.74E-03	5.70E-03	5.58E-03	5.23E-03	5.81E-03	6.46E-04	0.00E+00	0.00E+00	0.00E+00	0.00E+00	0.00E+00	0.00E+00	0.00E+00	0.00E+00	0.00000001
5-3-1-0	7.64E-03	6.41E-03	7.14E-03	5.91E-03	6.77E-03	7.66E-04	0.00E+00	0.00E+00	0.00E+00	0.00E+00	0.00E+00	0.00E+00	0.00E+00	0.00E+00	0.00000001
5-4-1-1	6.58E-03	6.62E-03	5.90E-03	7.00E-03	6.53E-03	4.56E-04	2.66E-02	2.08E-02	1.67E-02	2.74E-02	3.72E-02	2.20E-02	2.51E-02	7.11E-03	0.0009
5-5-0-2	0.00E+00	0.00E+00	0.00E+00	0.00E+00	0.00E+00	0.00E+00	8.78E-03	5.33E-03	5.38E-03	7.87E-03	9.28E-03	4.97E-03	6.93E-03	1.93E-03	0.0001
5-5-1-0	5.40E-03	5.38E-03	4.88E-03	5.65E-03	5.33E-03	3.21E-04	0.00E+00	0.00E+00	0.00E+00	0.00E+00	0.00E+00	0.00E+00	0.00E+00	0.00E+00	0.0000000001
5-6-1-1	0.00E+00	0.00E+00	0.00E+00	0.00E+00	0.00E+00	0.00E+00	1.01E-02	5.83E-03	5.19E-03	8.15E-03	1.02E-02	4.30E-03	7.29E-03	2.55E-03	0.0005
5-8-0-1	0.00E+00	0.00E+00	0.00E+00	0.00E+00	0.00E+00	0.00E+00	2.52E-02	3.09E-02	1.60E-02	1.91E-02	2.11E-02	1.15E-02	2.06E-02	6.85E-03	0.0004

Statistically significant *O* -Glycans in old female group with proteinuria (G1) and young female control group (G2)

Glycan	G1 156	G1 886	G1 887	G1 71112	Average relative abundance	Standard deviation	G2 175	G2 176	G2 177	G2 178	Average relative abundance	Standard deviation	p-value(Bonferroni correction)
1-1-0-1	1.14E-01	7.92E-02	8.35E-02	8.18E-02	8.97E-02	1.44E-02	2.40E-02	2.64E-02	2.03E-02	2.04E-02	2.28E-02	2.96E-03	0.0002
2-0-1-0	1.52E-02	1.29E-02	1.10E-02	1.11E-02	1.25E-02	1.72E-03	0.00E+00	0.00E+00	0.00E+00	0.00E+00	0.00E+00	0.00E+00	0.00002
2-1-0-0	0.00E+00	0.00E+00	0.00E+00	0.00E+00	0.00E+00	0.00E+00	3.32E-02	3.36E-02	3.54E-02	3.17E-02	3.35E-02	1.54E-03	0.00000001
2-1-0-1	8.67E-03	9.57E-03	7.96E-03	8.61E-03	8.70E-03	5.74E-04	0.00E+00	0.00E+00	0.00E+00	0.00E+00	0.00E+00	0.00E+00	0.0000002
2-1-0-2	3.71E-02	5.89E-02	5.94E-02	5.56E-02	5.27E-02	9.17E-03	1.02E-01	1.59E-01	1.38E-01	1.75E-01	1.44E-01	3.15E-02	0.002
2-4-1-2	1.79E-02	1.72E-02	1.57E-02	1.54E-02	1.65E-02	1.06E-03	0.00E+00	0.00E+00	0.00E+00	0.00E+00	0.00E+00	0.00E+00	0.0000002
2-4-2-2	9.40E-03	7.47E-03	7.80E-03	6.65E-03	7.83E-03	9.98E-04	0.00E+00	0.00E+00	0.00E+00	0.00E+00	0.00E+00	0.00E+00	0.00001
2-5-0-3	4.43E-03	5.05E-03	5.07E-03	5.11E-03	4.91E-03	2.80E-04	0.00E+00	0.00E+00	0.00E+00	0.00E+00	0.00E+00	0.00E+00	0.00000008
2-7-0-0	0.00E+00	0.00E+00	0.00E+00	0.00E+00	0.00E+00	0.00E+00	9.75E-03	1.23E-02	8.60E-03	6.44E-03	9.27E-03	2.44E-03	0.0003
3-0-0-0	0.00E+00	0.00E+00	0.00E+00	0.00E+00	0.00E+00	0.00E+00	1.17E-02	1.66E-02	2.03E-02	1.93E-02	1.69E-02	3.85E-03	0.0001
3-1-0-4	8.93E-03	8.76E-03	8.42E-03	8.26E-03	8.59E-03	2.68E-04	0.00E+00	0.00E+00	0.00E+00	0.00E+00	0.00E+00	0.00E+00	0.000000002
3-2-1-0	9.80E-03	9.56E-03	9.53E-03	6.29E-03	8.80E-03	1.45E-03	0.00E+00	0.00E+00	0.00E+00	0.00E+00	0.00E+00	0.00E+00	0.00004
3-2-2-0	4.58E-02	7.94E-02	6.23E-02	7.69E-02	6.61E-02	1.34E-02	0.00E+00	0.00E+00	0.00E+00	0.00E+00	0.00E+00	0.00E+00	0.0001
3-6-1-1	0.00E+00	0.00E+00	0.00E+00	0.00E+00	0.00E+00	0.00E+00	2.39E-02	2.02E-02	2.90E-02	1.95E-02	2.32E-02	4.31E-03	0.00004
4-3-2-1	0.00E+00	0.00E+00	0.00E+00	0.00E+00	0.00E+00	0.00E+00	2.82E-02	2.90E-02	4.66E-02	3.30E-02	3.42E-02	8.53E-03	0.0002
4-5-0-0	0.00E+00	0.00E+00	0.00E+00	0.00E+00	0.00E+00	0.00E+00	1.60E-02	1.76E-02	1.92E-02	1.72E-02	1.75E-02	1.33E-03	0.0000002
4-5-1-1	0.00E+00	0.00E+00	0.00E+00	0.00E+00	0.00E+00	0.00E+00	9.51E-03	8.75E-03	1.25E-02	7.74E-03	9.63E-03	2.06E-03	0.00008
5-3-1-0	0.00E+00	0.00E+00	0.00E+00	0.00E+00	0.00E+00	0.00E+00	5.97E-03	6.26E-03	7.63E-03	5.94E-03	6.45E-03	7.99E-04	0.000004
5-4-0-0	0.00E+00	0.00E+00	0.00E+00	0.00E+00	0.00E+00	0.00E+00	1.55E-02	1.51E-02	1.91E-02	1.32E-02	1.57E-02	2.45E-03	0.00001
5-4-1-0	0.00E+00	0.00E+00	0.00E+00	0.00E+00	0.00E+00	0.00E+00	7.02E-03	6.82E-03	9.77E-03	7.34E-03	7.74E-03	1.37E-03	0.00003
5-8-1-0	2.19E-03	2.04E-03	2.26E-03	1.78E-03	2.07E-03	1.83E-04	0.00E+00	0.00E+00	0.00E+00	0.00E+00	0.00E+00	0.00E+00	0.000001

Statistically significant *O* -Glycans in young female control group (G2) and young male control group (G4)

Glycan	G2 175	G2 176	G2 177	G2 178	Average relative abundance	Standard deviation	G4 175	G4 172	G4 1615A	G4 1615C	Average relative abundance	Standard deviation	p-value(Bonferroni correction)
1-1-0-1	2.40E-02	2.64E-02	2.03E-02	2.04E-02	2.28E-02	2.96E-03	0.00E+00	0.00E+00	0.00E+00	0.00E+00	0.00E+00	0.00E+00	0.000005
2-1-0-0	3.32E-02	3.36E-02	3.54E-02	3.17E-02	3.35E-02	1.54E-03	0.00E+00	0.00E+00	0.00E+00	0.00E+00	0.00E+00	0.00E+00	0.00000001
2-3-0-3	0.00E+00	0.00E+00	0.00E+00	0.00E+00	0.00E+00	0.00E+00	2.95E-03	2.37E-03	2.55E-03	3.11E-03	2.75E-03	3.44E-04	0.000004
2-3-1-2	0.00E+00	0.00E+00	0.00E+00	0.00E+00	0.00E+00	0.00E+00	1.06E-02	6.03E-03	8.08E-03	7.22E-03	7.99E-03	1.95E-03	0.0002
2-4-1-2	0.00E+00	0.00E+00	0.00E+00	0.00E+00	0.00E+00	0.00E+00	4.91E-03	4.15E-03	4.53E-03	3.35E-03	4.23E-03	6.69E-04	0.00001
2-4-2-1	0.00E+00	0.00E+00	0.00E+00	0.00E+00	0.00E+00	0.00E+00	1.03E-02	6.45E-03	8.83E-03	8.97E-03	8.64E-03	1.60E-03	0.00004
2-4-3-1	0.00E+00	0.00E+00	0.00E+00	0.00E+00	0.00E+00	0.00E+00	4.45E-03	7.08E-03	6.07E-03	8.19E-03	6.45E-03	1.59E-03	0.0002
2-5-0-0	0.00E+00	0.00E+00	0.00E+00	0.00E+00	0.00E+00	0.00E+00	8.49E-02	4.51E-02	6.37E-02	4.63E-02	6.00E-02	1.86E-02	0.0007
2-5-0-3	0.00E+00	0.00E+00	0.00E+00	0.00E+00	0.00E+00	0.00E+00	3.11E-03	3.12E-03	2.66E-03	3.63E-03	3.13E-03	3.99E-04	0.000004
2-5-1-3	0.00E+00	0.00E+00	0.00E+00	0.00E+00	0.00E+00	0.00E+00	8.78E-03	8.80E-03	7.53E-03	8.46E-03	8.39E-03	5.94E-04	0.0000001
2-6-0-0	0.00E+00	0.00E+00	0.00E+00	0.00E+00	0.00E+00	0.00E+00	2.03E-02	1.62E-02	1.90E-02	1.52E-02	1.77E-02	2.38E-03	0.000006
2-7-0-0	9.75E-03	1.23E-02	8.60E-03	6.44E-03	9.27E-03	2.44E-03	5.22E-02	5.87E-02	5.52E-02	5.58E-02	5.55E-02	2.65E-03	0.0000002
2-10-0-0	6.03E-02	6.79E-02	7.30E-02	6.21E-02	6.58E-02	5.81E-03	4.21E-02	4.03E-02	3.57E-02	3.60E-02	3.85E-02	3.18E-03	0.0002
3-0-0-0	1.17E-02	1.66E-02	2.03E-02	1.93E-02	1.69E-02	3.85E-03	3.31E-03	6.10E-03	3.77E-03	5.28E-03	4.62E-03	1.30E-03	0.0009
3-4-0-2	0.00E+00	0.00E+00	0.00E+00	0.00E+00	0.00E+00	0.00E+00	8.62E-03	1.04E-02	1.06E-02	9.80E-03	9.86E-03	9.02E-04	0.000001
3-5-1-1	0.00E+00	0.00E+00	0.00E+00	0.00E+00	0.00E+00	0.00E+00	1.10E-02	1.47E-02	1.43E-02	1.54E-02	1.38E-02	1.94E-03	0.000007
3-6-1-1	2.39E-02	2.02E-02	2.90E-02	1.95E-02	2.32E-02	4.31E-03	0.00E+00	0.00E+00	0.00E+00	0.00E+00	0.00E+00	0.00E+00	0.00004
4-3-0-2	0.00E+00	0.00E+00	0.00E+00	0.00E+00	0.00E+00	0.00E+00	3.28E-02	2.77E-02	2.76E-02	3.07E-02	2.97E-02	2.54E-03	0.0000004
4-4-1-1	0.00E+00	0.00E+00	0.00E+00	0.00E+00	0.00E+00	0.00E+00	2.18E-02	1.76E-02	1.99E-02	2.06E-02	2.00E-02	1.73E-03	0.0000004
4-4-2-0	0.00E+00	0.00E+00	0.00E+00	0.00E+00	0.00E+00	0.00E+00	8.47E-03	5.96E-03	6.78E-03	7.07E-03	7.07E-03	1.05E-03	0.00001
4-5-0-0	1.60E-02	1.76E-02	1.92E-02	1.72E-02	1.75E-02	1.33E-03	8.67E-02	6.00E-02	7.94E-02	5.86E-02	7.12E-02	1.41E-02	0.0003
4-5-1-1	9.51E-03	8.75E-03	1.25E-02	7.74E-03	9.63E-03	2.06E-03	0.00E+00	0.00E+00	0.00E+00	0.00E+00	0.00E+00	0.00E+00	0.00008
4-5-1-2	0.00E+00	0.00E+00	0.00E+00	0.00E+00	0.00E+00	0.00E+00	9.23E-03	8.06E-03	7.64E-03	8.88E-03	8.45E-03	7.28E-04	0.0000004
4-6-0-0	0.00E+00	0.00E+00	0.00E+00	0.00E+00	0.00E+00	0.00E+00	6.74E-03	5.70E-03	5.58E-03	5.23E-03	5.81E-03	6.46E-04	0.000002
5-4-1-0	7.02E-03	6.82E-03	9.77E-03	7.34E-03	7.74E-03	1.37E-03	0.00E+00	0.00E+00	0.00E+00	0.00E+00	0.00E+00	0.00E+00	0.00003
5-4-1-1	0.00E+00	0.00E+00	0.00E+00	0.00E+00	0.00E+00	0.00E+00	6.58E-03	6.62E-03	5.90E-03	7.00E-03	6.53E-03	4.56E-04	0.0000001
5-5-0-2	5.66E-03	4.89E-03	6.41E-03	5.55E-03	5.63E-03	6.24E-04	0.00E+00	0.00E+00	0.00E+00	0.00E+00	0.00E+00	0.00E+00	0.000002
5-5-1-0	1.46E-02	1.35E-02	1.70E-02	1.49E-02	1.50E-02	1.48E-03	5.40E-03	5.38E-03	4.88E-03	5.65E-03	5.33E-03	3.21E-04	0.00001
5-8-1-0	0.00E+00	0.00E+00	0.00E+00	0.00E+00	0.00E+00	0.00E+00	1.94E-03	2.11E-03	1.76E-03	2.75E-03	2.14E-03	4.30E-04	0.00006
